# Unveiling Long-Lived Hot-Electron Dynamics via Hyperbolic Meta-antennas

**DOI:** 10.1021/acs.nanolett.2c03922

**Published:** 2023-03-03

**Authors:** Rakesh Dhama, Mohsin Habib, Alireza R. Rashed, Humeyra Caglayan

**Affiliations:** Faculty of Engineering and Natural Science, Photonics, Tampere University, 33720 Tampere, Finland

**Keywords:** hyperbolic meta-antenna, ultrafast pump−probe spectroscopy, hot carriers, long-lived hot-electron dynamics, plasmon-modulated photoluminescence

## Abstract

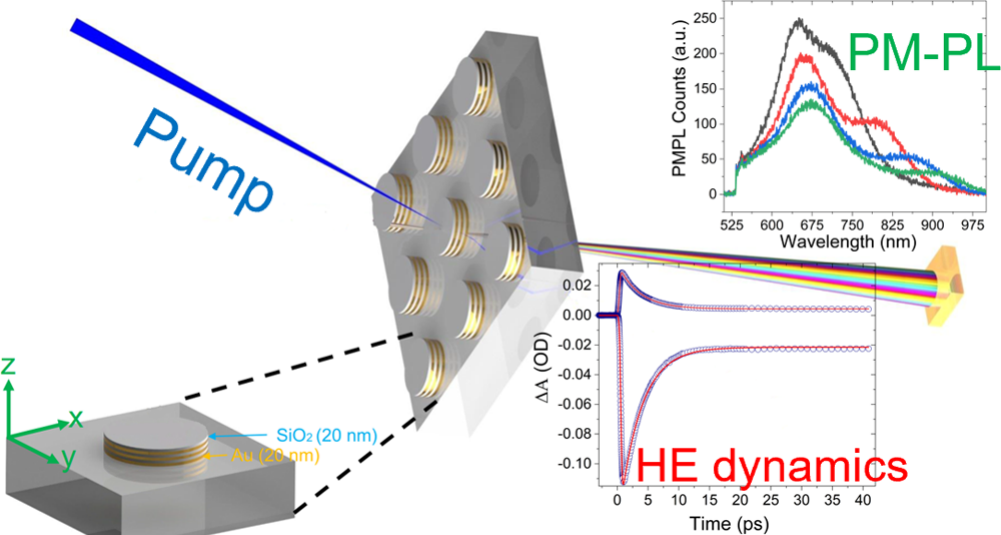

Conventional plasmonic nanoantennas enable scattering and absorption bands at the same wavelength region, making their utilization to full potential impossible for both features simultaneously. Here, we take advantage of spectrally separated scattering and absorption resonance bands in hyperbolic meta-antennas (HMA) to enhance the hot-electron generation and prolong the relaxation dynamics of hot carriers. First, we show that HMA enables extending plasmon-modulated photoluminescence spectrum toward longer wavelengths due to its particular scattering spectrum, in comparison to the corresponding nanodisk antennas (NDA). Then, we demonstrate that the tunable absorption band of HMA controls and modifies the lifetime of the plasmon-induced hot electrons with enhanced excitation efficiency in the near-infrared region and also broadens the utilization of the visible/NIR spectrum in comparison to NDA. Thus, the rational heterostructures designed by plasmonic and adsorbate/dielectric layers with such dynamics can be a platform for optimization and engineering the utilization of plasmon-induced hot carriers.

Plasmonic nanoantennas are well-known to enable extreme light confinement and enhanced electromagnetic field at the nanoscale. These properties provide a promising platform for harvesting and converting sunlight to chemical energy and driving photochemical reactions. Such plasmon-enhanced applications are attributed to the generation of hot carriers: hot electrons (HE) and hot holes (HH) through nonradiative plasmon decay.^1−3^

However, it is challenging to control and modify the dynamics of plasmon-generated hot carriers. HEs generated in plasmonic nanostructures suffer ultrashort life, low yield, and short mean free path due to ultrafast electron–electron scattering.^4,5^ These hinder the efficiency of plasmon-induced hot-electron transfer. So far, this issue has only been addressed by using different materials or optimizing the internal properties of the materials. Flexible control and tunability of the scattering and absorption channels are needed to fully control all aspects of hot-electron generation and the relaxation dynamics.

Additionally, plasmonic nanostructures accelerate electron–electron scattering at the excitation of interband transition and lead to the emission of particle plasmons (PPs) by hot carriers.^6,7^ In other words, excited d-band holes recombine nonradiatively with sp electrons, leading to the emission of PPs. These plasmons subsequently radiate, giving rise to photoluminescence (PL).^6^

Such particular PL is known as plasmon-modulated photoluminescence (PM-PL) and is directly correlated to the scattering spectra. PM-PL can be modulated by the plasmon resonances,^8^ which has characteristics of antiphotobleaching and antiphotoblinking, unlike conventional fluorophores, and is also thermally robust. These characteristics can open up potential applications, especially in the near-infrared (NIR) region.^9−12^ But due to the increased value of energy mismatch between the excitation laser and plasmon resonances, PM-PL spectra are only recorded up to visible wavelengths, which limits its applications. There have been several attempts to extend PM-PL in the NIR region by increasing the plasmonic nanoantenna size,^7^ changing the shapes of nanostructures,^8^ and using the roughened surfaces.^13,14^ However, PM-PL spectra have remained elusive in the NIR region through the conventional plasmonic nanoantennas to date.

Similarly, the traditional inorganic oxide semiconductors such as TiO_2_ and ZnO extensively used for photochemical reactions can only absorb in the ultraviolet region and only utilize a small part of the solar spectrum.^15^ Although plasmonic nanoantennas provide applications with improved features, they enhance scattering and absorption simultaneously at the same wavelength region. This limits these architectures to a specific application based on either the scattering or absorption process, especially in photoexcited systems.

Recently, nanoantennas obtained by nanostructuring bulk hyperbolic metamaterials with alternating layers of metal and dielectric have emerged as a unique platform to tune scattering, absorption, and local-field confinement.^16−18^ In particular, such hyperbolic nanoantennas can excite a super-radiant electric dipolar mode and a subradiant magnetic dipolar mode, enabling the modification (separation) of the scattering (radiative) and absorption (nonradiative) spectrum in the same architecture, leading to several unique applications.^19,20^

This work utilizes hyperbolic meta-antenna (HMA) type structures based on plasmonic metal/semiconductor or dielectric layers that can broaden the absorption spectra range and improve the lifetime of HEs as well as enhance the PM-PL excitation to a broader wavelength spectrum. We designed and fabricated a multilayer metal-dielectric (HMA) based on gold/silica stacking layers, enabling well-defined and separated scattering and absorption bands, while conventional plasmonic (gold) nanodisk antennas (NDA) of a thickness equivalent to that of total metal layers of an HMA are also realized as reference samples to distinguish the effect of multilayer HMA.

First, we investigate the plasmon-modulated photoluminescence (PM-PL) spectra in HMA as well as NDA systems. Second, we study the enhanced lifetime of energetic hot electrons excited by ultrafast photons at the interband transition in both types of structures. The effect of the separate absorption band in HMA on the generation and relaxation dynamics is also systematically investigated. This work explores the true potential of HMA with spectrally separated scattering and absorption regions. HMA brings dual functions such as PM-PL at longer wavelengths, attributed to its scattering band and an extended lifetime of hot electrons due to tunable absorption on a common platform, which is not possible in conventional plasmonic NDA.

To systematically investigate the features of the hot electrons in different plasmonic platforms, we designed and fabricated NDA with 60 nm thick gold and HMA based on multilayer metal-dielectric layers (3 bilayers of 20 nm gold and 20 nm SiO_2_). Figure 1a,b presents the measured transmission response of NDA and HMA for diameters from 100 to 180 nm with a step size of 20 nm, respectively. Transmission results show expectantly that the resonance in the transmission is red-shifted similarly for NDA and HMA as the diameter increases. However, the transmission results of HMAs differ, as two resonances are observed, one in a range similar to that of the NDA resonance while the other appears at longer wavelengths. Furthermore, Figure 1c shows cross-section field profiles in the *xz* plane of NDA at λ = 627 nm and HMA at λ = 662 and 847 nm. These field profiles reveal that the first mode of HMA is similar to that of NDA with strong field confinement on the metallic layers, whereas the second mode is much more confined inside the dielectric layers.

**Figure 1:**
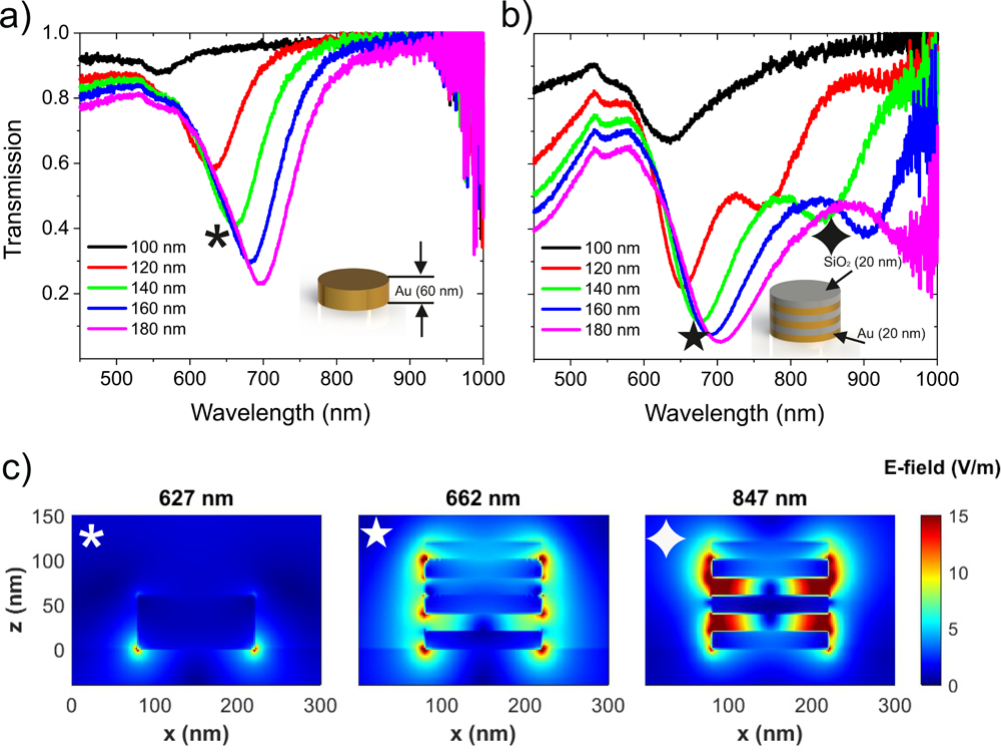
Measured transmission response of (a) NDAs and (b) HMAs with diameter sfrom 100 to 180 nm. (c) Electric field profiles in the *xz* plane of NDA with 140 nm diameter at λ = 627 nm and of HMA with 140 nm diameter at λ = 662 and 847 nm.

Finite-difference-time-domain (FDTD) simulations are performed to calculate the scattering and absorption of NDA and HMA structures with all diameters. As shown in Figure 2a,b, scattering is enhanced and red-shifted with an increase in the nanoantenna’s size.

**Figure 2:**
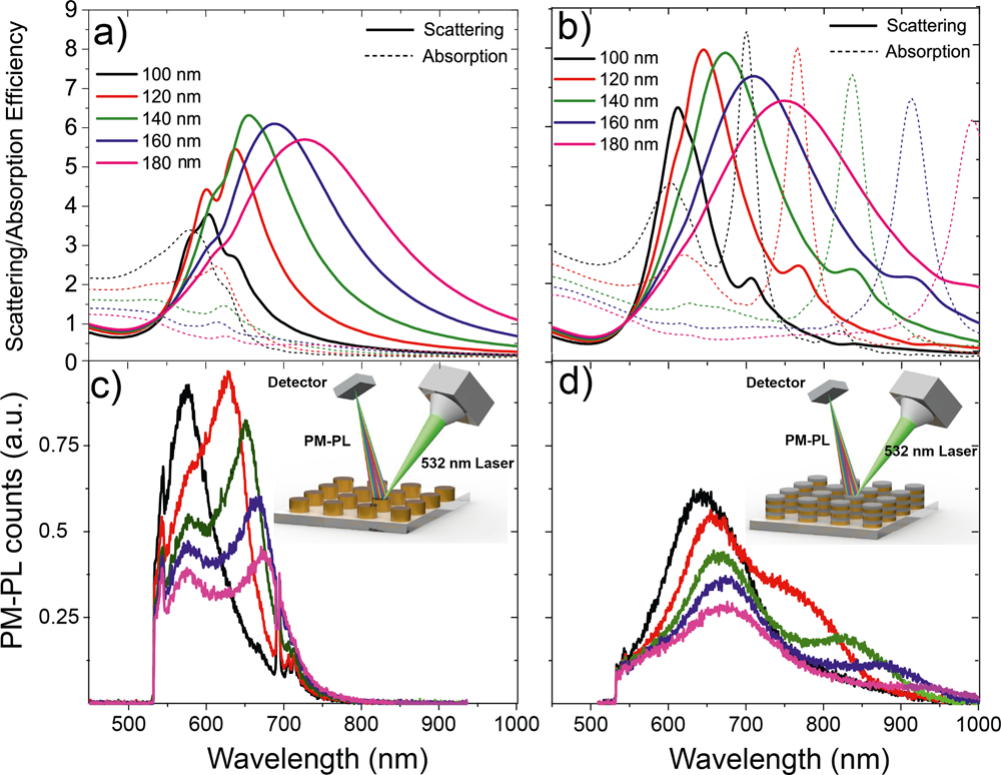
Calculated (a) scattering and absorption spectra of different sizes of NDA and (b) HMA structures. PM-PL spectra of (c) NDA structures and (d) HMAs with diameters from 100 to 180 nm excited by a linearly polarized green laser (λ = 532 nm).

Observation of higher scattering in HMAs as compared to NDA has been commonly achieved in simulations as well as experiments. This reflects the direct dependence of the nanostructure extinction on the predominant dipole mode. At the same time, the absorption of NDA structures is almost identical, while the absorption intensity and spectral position of HMAs are significantly modified with increasing nanoantenna diameter. The contribution of scattering to the total extinction (scattering/absorption) increases as absorption decreases for wavelengths more than 650 nm. The increase in the ratio of scattering to absorption with the nanostructure diameter is related to enhanced radiative damping in larger nanoparticles.^21−23^ The inclusion of 20 nm SiO_2_ between 20 nm slices of Au enables the modification of scattering and absorption and scattering, as shown in Figure 2b.

Thus, HMAs provide a potential solution for reshaping the absorption and scattering properties.

Once the optical properties of both plasmonic systems reveal that it is possible to modify the spectra using HMAs, we performed PM-PL measurements. PM-PL strictly follows the trends in the scattering of plasmonic structures and the energy mismatch between the excitation laser and plasmon resonance peak (Δ*E* = *E*_excitation_ – *E*_plasmon_).^7,8,24^ An increase in Δ*E* enables a significant decrease in PM-PL, which limits the fluorescence of metal nanostructures to the visible region. Figure 2c presents PM-PL measurements on NDA structures by exciting these structures using a linearly polarized green laser (λ = 532 nm) and reports the decrease in PM-PL as the diameter of nanodisks increases. An increase in the energy mismatch of the excitation wavelength and the plasmon resonance leads to a significant decrease in PM-PL. Therefore, for the smaller NDA structure, nonequilibrium electrons are highly populated to excite particle plasmons with a minimal value of energy mismatch and lead to the intensification of PM-PL at a shorter wavelength. Figure 2c further shows weaker photoluminescence spectra peaks at a longer wavelength, and PM-PL approaches a minimum value within the visible region for a larger NDA structure. When PM-PL measurements were performed on HMA systems under the same experimental conditions, broader PL spectra approached toward the NIR spectral region, which can be attributed to the reradiation of the scattering mode of the HMA structure. As presented in Figure 2d, interestingly, such PL enhancement in the NIR I (650–950 nm) region cannot be achieved in NDA due to the absence of such a peak (shoulder) in scattering spectra and an increase in energy mismatch. A PM-PL comparison for 120 nm diameter of both antennas clearly indicates the broadening of PL spectra up to 150 nm (toward the NIR region) in HMA with respect to NDA. Furthermore, PMPL spectra of 120 nm diameter HMA were also recorded as a function of incident laser intensity to confirm the linear nature of PM-PL (see Figure S3 in the Supporting Information). As the NIR wavelength region provides the maximum penetration of light through biological tissues, PM-PL in the NIR region is crucial to developing antiphotobleached fluorescent probes for imaging. At the same time, HMA also induces strong confinement of the electric field in the absorption band and increases the absorption efficiency to enhance the photothermal therapeutic capabilities.^25,26^

In photoexcited systems, coherent electron oscillation nonradiatively dephases and generates hot electrons (HEs) on a time scale ranging from 1 to 100 fs. HEs are generally those electrons that are not in thermal equilibrium with their immediate environment. They rapidly thermalize to a Fermi–Dirac distribution via different time scales, such as electron–electron scattering and electron–phonon scatterings (100 fs to 10 ps) that result in a higher lattice temperature followed by the slow dissipation of heat to the environment (100 ps to 10 ns).^27^ HEs have been utilized to trigger several chemical and physical phenomena. However, their novel applications are limited due to fast relaxation processes and low transfer efficiency from metals to acceptors. Thus, manipulating/engineering the spatial and temporal dynamics of HEs by particular plasmonic structures is key to developing exciting plasmon-induced hot carrier-based devices.

To understand the spatial and temporal dynamics of photogenerated HEs, an ultrafast transient absorption (TA) spectroscopic pump–probe setup has been employed in NDA and HMA systems of 120 nm diameter (*D*) with an array periodicity (*P*_*x*_ = *P*_*y*_) of 360 nm in transmission mode. The experimental method of TA experiments is discussed in detail in the Supporting Information.

Figure 3 shows the spatial and temporal dynamics of HEs due to interband transitions in the plasmonic NDA system at the excitation of 400 nm (3.1 eV) ultrafast pulses with a pump fluence of 255 μJ/cm^2^. Such excitation above threshold energy (2.38 eV) for interband transition in gold^28^ induces an electronic transition from the 5d band to the hybridized 6sp band, resulting in a transient electron population in the conduction band. In this context, a 3D surface panel represents a bird’s eye view of amplitude, wavelength, and time, and it is composed of several transient absorption spectra recorded at a succession of closely spaced time delays as presented in Figure 3a. Figure 3b reports TA spectra curves at 3 different time delays (1.92, 2.90, and 3.84 ps). TA spectra exhibit negative absorption (Δ*A*) (bleach region) centered at the plasmon band of NDA, along with two positive absorption (excessive absorption) bands at lower and higher energy with respect to the bleach region, as shown in Figure 3b. The positive transient absorption band around 550 nm is attributed to interband excitation of thermal electrons below the Fermi level.^29^ The negative absorption band (bleach region) peaked at around 640 nm, corresponding to the transition of electrons from lower energy levels to empty high-energy states above the Fermi level, while the positive transient spectral feature at around 670 nm appears due to the absorption of hot electrons and generation of interband excitation induced plasmons.^30,31^ The time-resolved decay profiles of the excessive absorption band at 577 nm and bleach region at 637 nm are extracted and fitted to almost similar times, 2.39 and 2.00 ps, respectively, as presented in Figure 3c,d, respectively. This means that time decay profiles of interband excitation and bleach region are similar, as they are both affected by the cooling of the hot electrons in the system.^29^

**Figure 3:**
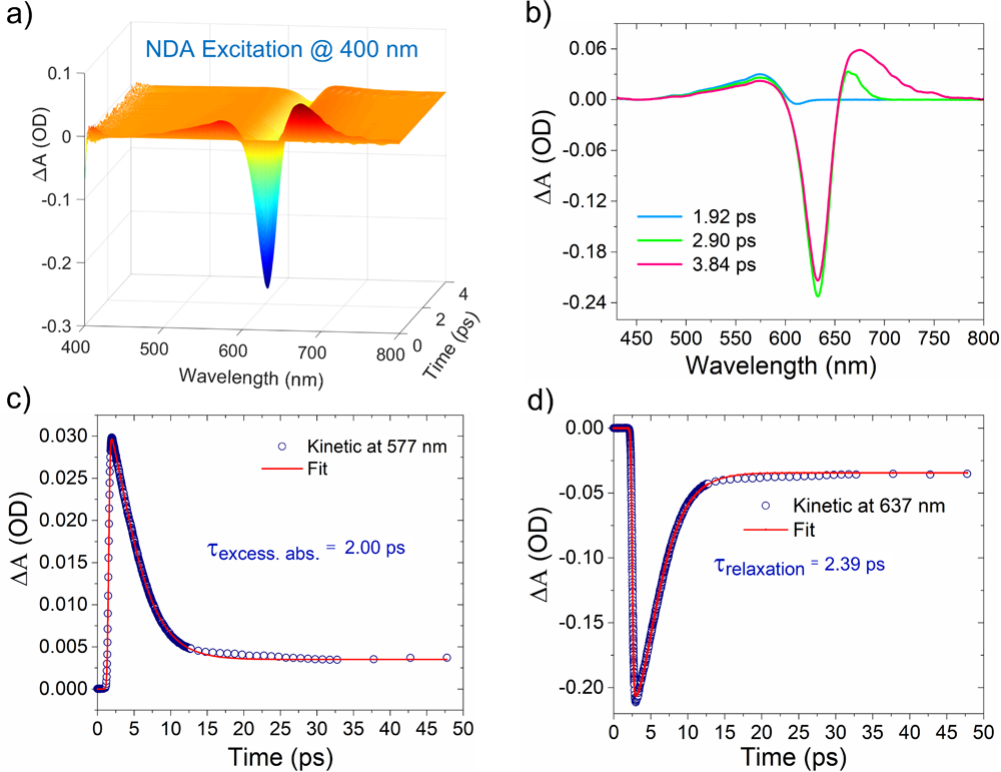
Transient absorption response of nanodisk antenna system (*D* = 120 nm, *P*_*x*_ = *P*_*y*_ = 360 nm) at interband excitation. (a) A 3D surface panel for Δ*A* spectra at different delay times for the NDA system at an excitation of 400 nm wavelength pump pulses. (b) Δ*A* spectra curves at specific time delays corresponding to the maximum absorption values. (c, d) Temporal dynamics of the excessive absorption band at 577 nm and bleach region at 637 nm for the NDA system.

Figure 4a presents 3D TA spectra as a function of wavelength and time for the HMA system using the same pump fluence (400 nm excitation wavelength) and similar experimental conditions used to understand the TA response of the NDA system. Figure 4b reports TA spectra curves from the bleach region’s peak of NDA and HMA systems to exhibit the broadening of transient response in HMA relative to NDA, while Figure 4c shows time-resolved decay profiles of the excessive absorption band at 527 nm and bleach region at 637 nm, which are fitted to very similar times of 3.45 and 3.50 ps, respectively.

**Figure 4:**
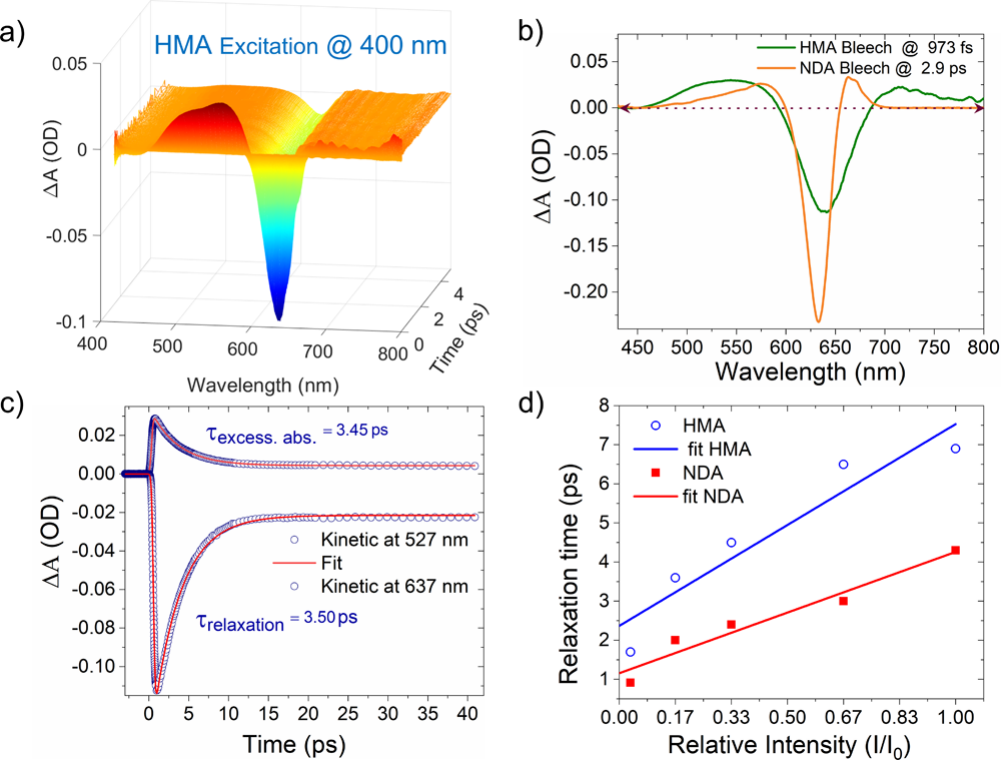
Transient absorption response of a hyperbolic meta-antenna system (*D* = 120 nm, *P*_*x*_ = *P*_*y*_ = 360 nm) and its comparison with a nanodisk antenna system at interband excitation. (a) A 3D surface panel for Δ*A* spectra at different delay times for the HMA system at an excitation of 400 nm wavelength pump pulses. (b) Extracted TA spectra curves of HMA and NDA systems at their bleach region’s maxima. (c) Temporal dynamics of the excessive absorption band at 527 nm and bleach region at 637 nm for the HMA system. (d) Experimental data and their best fit for a relaxation time of hot carriers at a 637 nm probe wavelength of the bleach region in NDA and HMA as a function of relative pump intensity.

To compare the ultrafast response of NDA and HMA systems, one can clearly see a broad transient response in HMA (Figure 4b) relative to the NDA system. This is attributed to the fact that an increase in the temperature enables a red shift and broadening of LSPR in Au nanostructures.^29,32^ Thus, the thermalization of HEs by pump excitation enables the transient red shift and broadening in the resonances of NDA and HMA. In particular, this phenomenon is more prominent in HMA, which can be attributed to slightly enhanced absorption across the whole visible range, enabling a significant hot-electron generation and relaxation in a broad range with respect to NDA. It is also noteworthy that hot-electron relaxation time (bleach region) in HMA is almost twice (3.5 ps) that of NDA (2.0 ps) under the interband excitation (3.1 eV) at the same pump power. However, it is well-known that the relaxation dynamics of HEs depend on the amount of absorbed pump energy (*E*_abs_) by the plasmonic structure, and higher absorbed energy enables a longer-lived relaxation time.^33−35^ Therefore, to compare relaxation dynamics for two different samples, the relaxation time of hot carriers must be obtained by extrapolating the relaxation time to *E*_abs_ = 0, where relaxation time is independent of pump energy.^33^ It is clear from the linear fit of observed values as shown in Figure 4d that HMA reports an elongated relaxation time (2.3 ps) in comparison to the lifetime (1.15 ps) of NDA at zero absorption of pump energy. However, at the recorded lowest pump fluence (120 μJ/cm^2^), HMA exhibits a 1.7 ps relaxation time of hot carriers in comparison to that of 0.91 ps in NDA, which is an increase of almost 2 times.

Hot-electron injections from metals into an adsorbate/semiconductor have been extensively reported in Au/TiO_2_,^15,27,36,37^ Au/SiC,^38^ and Au/Si interfaces.^3^ Due to the lower values of the Schottky barrier (ϕ_SB_) in these systems, a fraction of hot electrons cross the potential barrier to prolong their lifetime and trigger the chemical reactions in the semiconductor/adsorbate before the recombination with holes. In contrast, the remaining HEs that are unable to cross the potential barrier have relatively short lifetime and cannot participate in photochemical transformations. Moreover, HEs significantly lose the energy to travel across the barrier, affecting photocatalysis efficiency on plasmonic/semiconductor heterostructures.

Thus, efficient HE generation and its accumulation for HE flux enhancement at the region of interest is a crucial issue that needs to be addressed for improved performance and novel hot-electron-based mechanisms. It has been shown that the rate of plasmon-induced H_2_ dissociation on Au NPs based on the dielectric SiO_2_^39^ is enhanced by 2 orders of magnitude than that observed on equivalently prepared Au NPs on TiO_2_.^40^ This enhancement in the dissociation efficiency of H_2_ is attributed to a large number of HEs present on the Au/SiO_2_ interface as HE injection into the wide-band-gap dielectric SiO_2_ (9 eV) is not allowed and results in strong HE flux at the interface to enhance the chemical reactions in comparison to Au/TiO_2_ (band gap 3.1 eV). Such an improvement in electron flux also leads to an elongation of the lifetime of HEs. Therefore, the accumulation of excited hot electrons at the interfaces of gold/silica and the increase in HE flux due to enhanced absorption of pump energy in HMA explains the elongated HE’s relaxation lifetime as compared to that of NDA.

Additionally, the HMA system advances the utilization of the spectrum due to the separate absorption band in NIR (see Figure 2c) and excites HEs with a further elongated time at the excitation of NIR pump pulses. Under similar circumstances, NDA does not exhibit any transient response due to the almost negligible absorption in the NIR region (see the blue dashed curve in Figure 2a). To investigate the effect of the induced absorption band on the temporal dynamics of HEs created in HMA, we have performed TA experiments on an HMA (120 nm diameter) system excited by a 780 nm wavelength (in-resonance) and 730 nm (out-of-resonance) ultrafast pulse pump fluence of 255 μJ/cm^2^. Figure 5a,b reports time-resolved decay profiles of the excessive absorption band at 547 nm and bleach region at 664 nm, fitted to time decays of 5.0 and 6.7 ps, respectively, when excited in the so-called *in-resonance band* (780 nm wavelength pump pulses), as clearly indicated in the inset of Figure 5a. Similarly, Figure 5c,d shows time-resolved decay profiles of the excessive absorption band and bleach region at the excitation of the *out-of-resonance band* (730 nm wavelength pump pulses), as shown in the inset of Figure 5c. The fitted time decays are 3.5 and 3.0 ps, respectively, for the different observed wavelengths. The results clearly confirm that absorption efficiency and induced field confinement of the plasmonic structures are also crucial factors in prolonging the lifetime of HEs.

**Figure 5:**
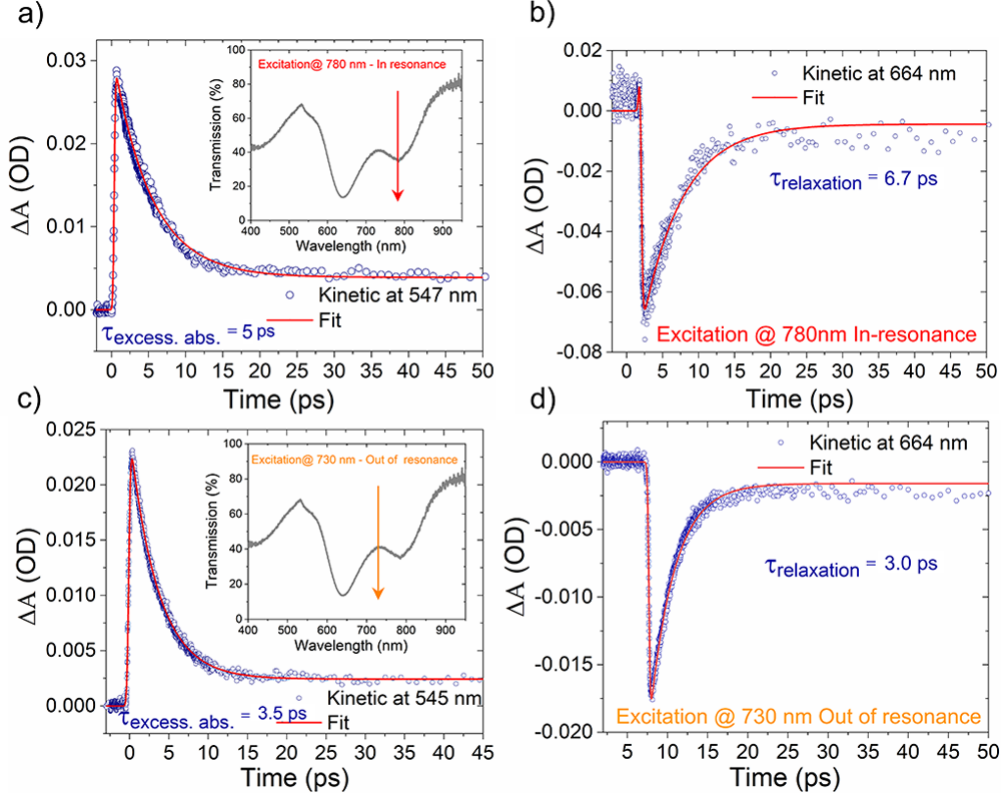
(a, b) Temporal dynamics of the excessive absorption band at 547 nm and bleach region at 664 nm for the HMA system on excitation by 780 nm wavelength pump pulses in the center of the separate absorption band, as indicated in the inset in (a). (c, d) Temporal dynamics of the excessive absorption band at 545 nm and bleach region at 664 nm for HMA system on excitation by 730 nm wavelength pump pulses which are out of the separate absorption band, as shown in the inset of (c).

The enhanced absorption feature of HMA at a separated absorption band and strong induced field enables an elongated HE lifetime at the in-resonance excitation. While HEs have a short lifetime at out-of-resonance excitation (730 nm) due to lower absorption and weaker field by changing the thickness and composition of plasmonic and dielectric layers, HMA can also efficiently generate HEs from UV to deep-NIR wavelength ranges. To further confirm the role of absorption efficiency of the HMA in HE generation, an additional TA experiment has been performed on HMA (160 nm diameter) on excitation by 780 nm NIR pump pulses with the same pump power (see Figure S3 in the Supporting Information). This system does not exhibit any transient response due to low absorption at the 780 nm probe region (see the blue dashed curve in Figure 2b).

In conclusion, we have exploited the separate scattering and absorption bands in HMA for two specific purposes and compared their characteristics with those of common plasmonic systems at the same time. Ultrafast transient absorption results reveal that the lifetime of HEs in HMA is elongated due to the enhanced HE flux at gold/silica interfaces compared to NDA at interband excitation. HMA also enables photoinduced HEs with an elongated lifetime at NIR wavelengths due to the presence of a separate absorption band, which is not possible in NDA. Such architectures like that of HMA successfully broaden the utilization of the solar spectrum compared to NDA and can be designed and optimized to excite a specific lifetime of HEs on demand.

Moreover, based on the PM-PL spectra in HMA and NDA, the PM-PL spectra of HMA extend to longer NIR wavelengths due to the presence of an additional peak (shoulder) in HMA’s scattering spectra. NDA does not exhibit this feature, and PL from gold nanodisks is limited to the visible range. Therefore, HMA offers enhanced functions through a single platform (photoexcitation of electrons in the structure), exploiting scattering spectra to enable extended PM-PL spectra and broadening the absorption spectra with an elongated HE lifetime due to the spectrally separated absorption band. Thus, such functionalized structures can open up a strategy to excite HEs with a specific or engineered lifetime for all-optical control of photocatalysis.

## Supporting Information Available

Additional details of materials and methods such as fabrication of NDA and HMA, numerical simulations for calculated transmission, E-field distribution and effective permittivity of HMA, experimental transmission, and power-dependent PM-PL measurements and additional details on methods used for transient absorption spectroscopy measurements and the transient response of 160 nm diameter HMA at the excitation of NIR pump pulses (PDF)

## References

[ref1] Choi J. Y.; Jeong D.; Lee S. J.; Kang D.-g.; Kim S. K.; Nam K. M.; Song H. Engineering Reaction Kinetics by Tailoring the Metal Tips of Metal–Semiconductor Nanodumbbells. Nano Lett. 2017, 17, 5688–5694. 10.1021/acs.nanolett.7b02582. 28850244

[ref2] Han C.; Li S.-H.; Tang Z.-R.; Xu Y.-J. Tunable plasmonic core–shell heterostructure design for broadband light driven catalysis. Chemical science 2018, 9, 8914–8922. 10.1039/C8SC04479A. 30746116 PMC6335626

[ref3] Knight M. W.; Sobhani H.; Nordlander P.; Halas N. J. Photodetection with active optical antennas. Science 2011, 332, 702–704. 10.1126/science.1203056. 21551059

[ref4] Wu K.; Chen J.; McBride J. R.; Lian T. Efficient hot-electron transfer by a plasmon-induced interfacial charge-transfer transition. Science 2015, 349, 632–635. 10.1126/science.aac5443. 26250682

[ref5] Ratchford D. C. Plasmon-induced charge transfer: Challenges and outlook. ACS Nano 2019, 13, 13610–13614. 10.1021/acsnano.9b08829. 31809010

[ref6] Dulkeith E.; Niedereichholz T.; Klar T.; Feldmann J.; Von Plessen G.; Gittins D.; Mayya K.; Caruso F. Plasmon emission in photoexcited gold nanoparticles. Phys. Rev. B 2004, 70, 205424 10.1103/PhysRevB.70.205424.

[ref7] Rashed A. R.; Habib M.; Das N.; Ozbay E.; Caglayan H. Plasmon-modulated photoluminescence enhancement in hybrid plasmonic nano-antennas. New J. Phys. 2020, 22, 093033 10.1088/1367-2630/abaf69.

[ref8] Hu H.; Duan H.; Yang J. K.; Shen Z. X. Plasmon-modulated photoluminescence of individual gold nanostructures. ACS Nano 2012, 6, 10147–10155. 10.1021/nn3039066. 23072661

[ref9] He H.; Xie C.; Ren J. Nonbleaching fluorescence of gold nanoparticles and its applications in cancer cell imaging. Analytical chemistry 2008, 80, 5951–5957. 10.1021/ac8005796. 18590338

[ref10] Tong L.; Cobley C. M.; Chen J.; Xia Y.; Cheng J.-X. Bright Three-Photon Luminescence from Gold/Silver Alloyed Nanostructures for Bioimaging with Negligible Photothermal Toxicity. Angew. Chem., Int. Ed. 2010, 49, 3485–3488. 10.1002/anie.201000440. PMC3009572 20544899

[ref11] Hirsch L. R.; Stafford R. J.; Bankson J. A.; Sershen S. R.; Rivera B.; Price R. E.; Hazle J. D.; Halas N. J.; West J. L. Nanoshell-mediated near-infrared thermal therapy of tumors under magnetic resonance guidance. JL Proc. Natl. Acad. Sci. USA 2003, 100, 13549–13554. 10.1073/pnas.2232479100. PMC263851 14597719

[ref12] Lu G.; Hou L.; Zhang T.; Liu J.; Shen H.; Luo C.; Gong Q. Plasmonic sensing via photoluminescence of individual gold nanorod. J. Phys. Chem. C 2012, 116, 25509–25516. 10.1021/jp309450b.

[ref13] Boyd G.; Yu Z.; Shen Y. Photoinduced luminescence from the noble metals and its enhancement on roughened surfaces. Phys. Rev. B 1986, 33, 7923 10.1103/PhysRevB.33.7923. 9938182

[ref14] Wan A.; Wang T.; Yin T.; Li A.; Hu H.; Li S.; Shen Z. X.; Nijhuis C. A. Plasmon-modulated photoluminescence of single gold nanobeams. ACS Photonics 2015, 2, 1348–1354. 10.1021/acsphotonics.5b00341.

[ref15] Zhang Y.; He S.; Guo W.; Hu Y.; Huang J.; Mulcahy J. R.; Wei W. D. Surface-plasmon-driven hot electron photochemistry. Chem. Rev. 2018, 118, 2927–2954. 10.1021/acs.chemrev.7b00430. 29190069

[ref16] Maccaferri N.; Zhao Y.; Isoniemi T.; Iarossi M.; Parracino A.; Strangi G.; De Angelis F. Hyperbolic meta-antennas enable full control of scattering and absorption of light. Nano Lett. 2019, 19, 1851–1859. 10.1021/acs.nanolett.8b04841. 30776244

[ref17] Zhao Y.; Hubarevich A.; Iarossi M.; Borzda T.; Tantussi F.; Huang J.-A.; De Angelis F. Hyperbolic Nanoparticles on Substrate with Separate Optical Scattering and Absorption Resonances: A Dual Function Platform for SERS and Thermoplasmonics. Advanced Optical Materials 2021, 9, 2100888 10.1002/adom.202100888.

[ref18] Indukuri S. R. K.; Frydendahl C.; Bar-David J.; Mazurski N.; Levy U. WS2Monolayers Coupled to Hyperbolic Metamaterial Nanoantennas: Broad Implications for Light–Matter-Interaction Applications. ACS Applied Nano Materials 2020, 3, 10226–10233. 10.1021/acsanm.0c02186.

[ref19] Maccaferri N.; Zilli A.; Isoniemi T.; Ghirardini L.; Iarossi M.; Finazzi M.; Celebrano M.; De Angelis F. Enhanced nonlinear emission from single multilayered metal–dielectric nanocavities resonating in the near-infrared. ACS Photonics 2021, 8, 512–520. 10.1021/acsphotonics.0c01500.

[ref20] Kuttruff J.; Gabbani A.; Petrucci G.; Zhao Y.; Iarossi M.; Pedrueza-Villalmanzo E.; Dmitriev A.; Parracino A.; Strangi G.; De Angelis F.; et al. Magneto-optical activity in nonmagnetic hyperbolic nanoparticles. Physical review letters 2021, 127, 217402 10.1103/PhysRevLett.127.217402. 34860084

[ref21] Sönnichsen C.; Franzl T.; Wilk T.; Von Plessen G.; Feldmann J. Plasmon resonances in large noble-metal clusters. New J. Phys. 2002, 4, 93 10.1088/1367-2630/4/1/393.

[ref22] Scaffardi L. B.; Pellegri N.; De Sanctis O.; Tocho J. O. Sizing gold nanoparticles by optical extinction spectroscopy. Nanotechnology 2005, 16, 158 10.1088/0957-4484/16/1/030.

[ref23] Sönnichsen C.; Franzl T.; Wilk T.; von Plessen G.; Feldmann J.; Wilson O.; Mulvaney P. Drastic reduction of plasmon damping in gold nanorods. Physical review letters 2002, 88, 077402 10.1103/PhysRevLett.88.077402. 11863939

[ref24] Ngoc L. L. T.; Wiedemair J.; van den Berg A.; Carlen E. T. Plasmon-modulated photoluminescence from gold nanostructures and its dependence on plasmon resonance, excitation energy, and band structure. Opt. Express 2015, 23, 5547–5564. 10.1364/OE.23.005547. 25836787

[ref25] Roper D. K.; Ahn W.; Hoepfner M. Microscale heat transfer transduced by surface plasmon resonant gold nanoparticles. J. Phys. Chem. C 2007, 111, 3636–3641. 10.1021/jp064341w. PMC2583113 19011696

[ref26] Zhao Y.; Hubarevich A.; Iarossi M.; Borzda T.; Tantussi F.; Huang J.-A.; De Angelis F. Hyperbolic Nanoparticles on Substrate with Separate Optical Scattering and Absorption Resonances: A Dual Function Platform for SERS and Thermoplasmonics. Advanced Optical Materials 2021, 9, 2100888 10.1002/adom.202100888.

[ref27] Brongersma M. L.; Halas N. J.; Nordlander P. Plasmon-induced hot carrier science and technology. Nature Nanotechnol. 2015, 10, 25–34. 10.1038/nnano.2014.311. 25559968

[ref28] Schoenlein R.; Lin W.; Fujimoto J.; Eesley G. Femtosecond studies of nonequilibrium electronic processes in metals. Phys. Rev. Lett. 1987, 58, 1680 10.1103/PhysRevLett.58.1680. 10034506

[ref29] Wang Y.; Shi H.; Shen L.; Wang Y.; Cronin S. B.; Dawlaty J. M. Ultrafast dynamics of hot electrons in nanostructures: distinguishing the influence on interband and plasmon resonances. ACS Photonics 2019, 6, 2295–2302. 10.1021/acsphotonics.9b00793.

[ref30] Logunov S.; Ahmadi T.; El-Sayed M.; Khoury J.; Whetten R. Electron dynamics of passivated gold nanocrystals probed by subpicosecond transient absorption spectroscopy. J. Phys. Chem. B 1997, 101, 3713–3719. 10.1021/jp962923f.

[ref31] Zhang X.; Huang C.; Wang M.; Huang P.; He X.; Wei Z. Transient localized surface plasmon induced by femtosecond interband excitation in gold nanoparticles. Sci. Rep. 2018, 8, 1–7. 10.1038/s41598-018-28909-6. 30002475 PMC6043523

[ref32] Yeshchenko O.; Bondarchuk I.; Gurin V.; Dmitruk I.; Kotko A. Temperature dependence of the surface plasmon resonance in gold nanoparticles. Surf. Sci. 2013, 608, 275–281. 10.1016/j.susc.2012.10.019.

[ref33] Hodak J.; Martini I.; Hartland G. V. Ultrafast study of electron–phonon coupling in colloidal gold particles. Chemical physics letters 1998, 284, 135–141. 10.1016/S0009-2614(97)01369-9.

[ref34] Della Valle G.; Conforti M.; Longhi S.; Cerullo G.; Brida D. Real-time optical mapping of the dynamics of nonthermal electrons in thin gold films. Phys. Rev. B 2012, 86, 155139 10.1103/PhysRevB.86.155139.

[ref35] Hartland G. V. Optical studies of dynamics in noble metal nanostructures. Chem. Rev. 2011, 111, 3858–3887. 10.1021/cr1002547. 21434614

[ref36] Liu L.; Ouyang S.; Ye J. Gold-nanorod-photosensitized titanium dioxide with wide-range visible-light harvesting based on localized surface plasmon resonance. Angew. Chem. 2013, 125, 6821–6825. 10.1002/ange.201300239. 23666880

[ref37] Clavero C. Plasmon-induced hot-electron generation at nanoparticle/metal-oxide interfaces for photovoltaic and photocatalytic devices. Nat. Photonics 2014, 8, 95–103. 10.1038/nphoton.2013.238.

[ref38] Hao C.-H.; Guo X.-N.; Pan Y.-T.; Chen S.; Jiao Z.-F.; Yang H.; Guo X.-Y. Visible-light-driven selective photocatalytic hydrogenation of cinnamaldehyde over Au/SiC catalysts. J. Am. Chem. Soc. 2016, 138, 9361–9364. 10.1021/jacs.6b04175. 27403658

[ref39] Mukherjee S.; Zhou L.; Goodman A. M.; Large N.; Ayala-Orozco C.; Zhang Y.; Nordlander P.; Halas N. J. Hot-electron-induced dissociation of H2 on gold nanoparticles supported on SiO2. J. Am. Chem. Soc. 2014, 136, 64–67. 10.1021/ja411017b. 24354540

[ref40] Mukherjee S.; Libisch F.; Large N.; Neumann O.; Brown L. V.; Cheng J.; Lassiter J. B.; Carter E. A.; Nordlander P.; Halas N. J. Hot electrons do the impossible: plasmon-induced dissociation of H2 on Au. Nano Lett. 2013, 13, 240–247. 10.1021/nl303940z. 23194158

