# Supplementary material for: Unveiling Long-Lived Hot-Electron Dynamics via Hyperbolic Meta-antennas
Source: Nano Lett. 2023 Mar 3;23(8):3122–7. doi: 10.1021/acs.nanolett.2c03922 (PMC10141405; doi:10.1021/acs.nanolett.2c03922)
Supplement: Supplementary file 1 — nl2c03922_si_001.pdf [file nl2c03922_si_001.pdf]

Supplementary Information
Unveiling Long-Lived Hot Electron dynamics via hyperbolic meta-antennas

Rakesh Dhama, Mohsin Habib, Alireza R. Rashed, and Humeyra Caglayan*

Faculty of Engineering and Natural Science, Photonics, Tampere University, 33720 Tampere, Finland

E-mail: humeyra.caglayan@tuni.fi

Methods

Numerical simulations

We use the commercially available software, ANSYS Lumerical FDTD Solutions, for 3D electromagnetic simulations of scattering, absorption and transmission, and field profiles of the samples. The refractive index values are taken from experimental data available in the literature to model Au,[1] and SiO_2.[2] To simulate the scattering and absorption, the boundary conditions (BCs) are set to perfect match layers (PMLs) in all directions. We use a Total-Field Scattered-Field (TFSF) source of wavelengths 450-1000 nm. The fields inside the source are the sum of incident fields and scattered fields, while only the scattered fields are visible outside the source. The analysis group calculates the absorption cross-section using the optical theorem. The uniform meshing of 2 nm was used to calculate scattering and absorption.

For transmission calculations, the unit cell of size 440 nm is illuminated by a linearly x-polarized plane wave source of wavelengths 450-1000 nm. The boundary conditions (BCs) are set to periodic along x, y axes, and perfectly matched layers (PMLs) in the direction propagation z. Conformal meshing is used in simulations, while a finer mesh constraint of 2 nm is employed in the region enclosing the NDA and HMA to get a better resolution. Similarly, cross-sectional E_z and H_y fields are calculated at single wavelength values along different planes.

Fabrication

We fabricated the NDA using standard electron beam lithography (EBL), followed by the metal deposition and lift-off process. However, for HMA, we first deposited an alternative layer of Au/SiO_2, then performed the EBL followed by the deposition of nickel (Ni) as a mask. Next, the lift-off process and reactive ion etching (RIE) of the unwanted layer have been done. The 100x100 μm^2 write field is used to create the NDAs with the area dose of 300 $\mu C/cm^2$. The exposed samples are developed for 60 sec in 1:3 Metylisobutylketon(MIBK): IPA solution and 30 sec in IPA to stop the development process. The developed samples are loaded in the electron beam evaporation deposition chamber to deposit 1.5 nm of Ti and 60 nm of Au. The S-1165 remover is used to lift off the unwanted Au.

For HMA, the cleaned glass samples are coated with alternative layers of Au and SiO_2 using an electron-beam evaporator. For better adhesion, 1 nm of Ti is used before each layer. Once the multilayers are ready, a nanodisk array is formed using similar e-beam lithography parameters to NDA. The developed samples in this case are coated with 15 nm Ni. The lift-off is done to remove unwanted metal from the sample and form Ni disks that serve as a mask. To transfer these pattering to multilayers and form HMA, RIE is performed using oxford instruments plasma technology machine. Argon and Fluoroform (CHF_3) gases are used in the process. The flow of both gases was 25 sccm, radio frequency (RF) power 200 W, and pressure of 30 mTorr, and the etching time was set to 15 minutes. The etching time

was first optimized for multilayers and Ni case and 15 min.

Optical characterization

Transmission Measurements

Transmission spectra are measured using a microscope from WiTec (alpha300 R- Confocal Raman Imaging). The samples are excited with a broadband light source (Energetiq EQ-99XFC LDLS, spectrum 190 nm to 2100 nm). The optical pump beam is focused on the sample surface using a 20x objective (Zeiss NA=0.4) in normal incidence. To detect the transmission spectra, a 50x objective (Zeiss NA=0.75) is placed at the back focal plane to collect transmitted light in a normal direction. The collected light is coupled to an optical fiber connected to a spectrometer (Ocean Optics Flame, detection range 400 nm - 900 nm). A neutral density filter of 2 (AR coated from Thorlabs) is placed in the beam path to reduce the beam spot size and then focused on the back focal plane. We first measure the transmission spectra from the glass substrate. Then, we measure the transmission spectrum of the meta-antenna arrays normalized to the glass spectrum.

PM-PL Measurements

PM-PL measurements on HMA and NDA hybrid structures are performed on a multi-functional WITec alpha300C confocal Raman microscopy system. The photoluminescence spectra of the control and main samples are acquired utilizing a VIS-NIR Flame detector (350–1000 nm) provided by Ocean Optics with an integration time of 300 ms. The samples are excited with a 532 nm CW laser, and the PL signals are guided to the detector through a 50×Zeiss EC 'Epiplan' DIC objective (NA = 0.75, WD =1.0 mm).

Transient absorption spectroscopy measurements

Ultrafast time-resolved pump-probe spectroscopy was performed using an amplified Ti: sapphire laser system equipped with an optical parametric amplifier (OPA). This system pro-

duced 100 fs pulses at 1.00 kHz with a center wavelength of 800 nm. Most of the output (90%) was directed to the OPA to generate tunable pump pulses in the UV-Visible to near-infrared spectral regions to excite interband and plasmonic resonances, respectively. The remaining 10% of output power travels through a delay-line to enable controlled time difference between pump and probe pulses and converts into a broadband probe beam to interrogate the sample at the normal incidence in transmission mode. At the same time, The chopper-modulated pump pulse is spectrally as well as temporally overlapped with the probe beam on the sample. At the same time, the detector is triggered to detect every probe pulse and calculate the absorption spectrum. Repetition rates of the pump and probe beams turn out to be 500 Hz and 1 kHz, respectively. Therefore, the effect of the pump beam will be observed only in one of the two consecutive probe beams.

Calculated Transmission Spectra and E-field distributions

Figure S1 presents the transmission response of NDA and HMA. E-field profile in the xy plane of NDA is calculated for only one wavelength (λ= 627 nm), and E-field profile of HMA for two different wavelengths corresponding to the scattering and absorption maximum (λ= 662 nm and λ= 847 nm). The field profile shows that in both of the structures the field shows the dipolar mode effect. Moreover, the field is more confined in HMA structures compared to the NDA. This is also in agreement with the high scattering properties of HMA structures. The radiative mode has a strong dipolar field in x-axis and will result in strong interactions.

Effective permittivity of Hyperbolic Metamaterials

The optical properties of thin film based hyperbolic metamaterials (HMMs) are modelled by the effective approximation (EMA),[3] where in-plane isotropic/parallel components are defined as $\varepsilon_{xx} = \varepsilon_{yy} = \varepsilon_{\parallel}$. The third component which is out of the plane (perpendicular component) is defined as $\varepsilon_{zz} = \varepsilon_{\perp}$

The parallel and perpendicular components of permittivity of a medium composed of

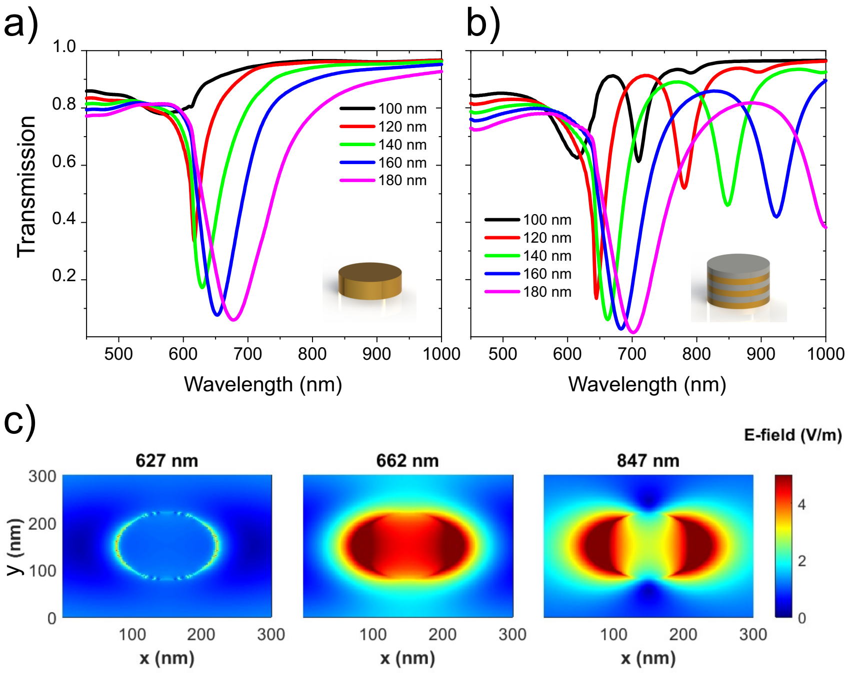

Figure S1: The calculated transmission response of (a) NDAs, (b) HMAs with a diameter from 100 to 180 nm. (c) Electric field profiles in the xy plane of NDA with 140 nm diameter at λ= 627 nm, 140 nm diameter HMA at λ= 662 nm and 847 nm.

alternating thin layers of metal and dielectric are defined as follows:

$$\varepsilon_{\parallel} = \rho\varepsilon_m + (1-\rho)\varepsilon_d \tag{1}$$

$$\varepsilon_{\perp} = \frac{\varepsilon_m \varepsilon_d}{\rho\varepsilon_d + (1-\rho)\varepsilon_m} \tag{2}$$

where $\rho = t_m/(t_m+t_d)$ is the metal filling fraction, with $t_{m(d)}$ being the thickness of the metal (dielectric) layer, and ε_m and ε_d are permittivity values of metal and dielectric, respectively. Figure S2 presents the $\varepsilon_{\parallel}$ and $\varepsilon_{\perp}$ components of HMA with thickness of 20/20 nm for Au/SiO_2.

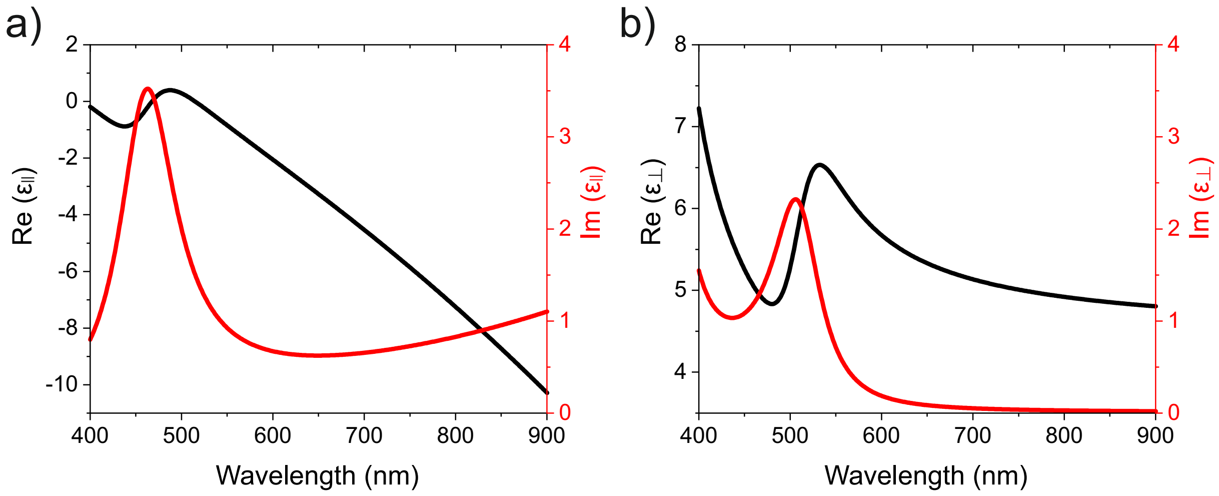

Figure S2: The (a) parallel and (b) perpendicular components of permittivity for HMA calculated using EMA.

Power dependent plasmon modulated photoluminescence (PMPL) of HMA

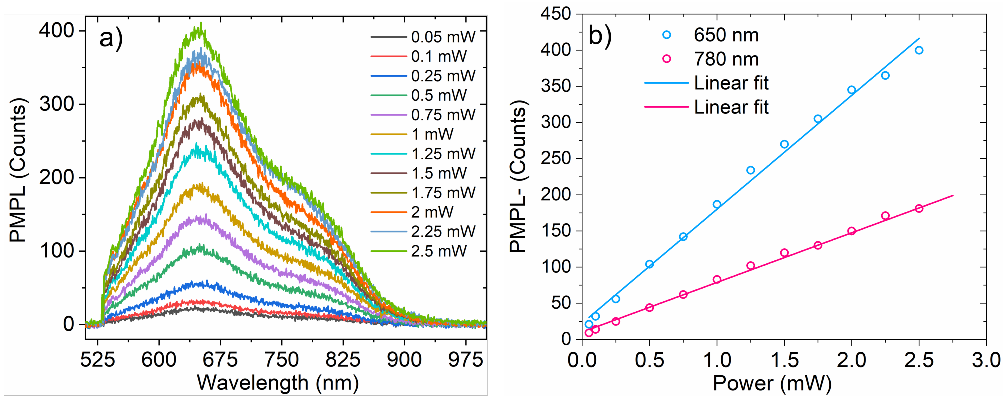

Figure S3: (a) PMPL intensities of HMA (Diameter = 120 nm and Period = 360 nm) vs. incident laser intensity when excited by 532 nm CW laser. (b) Linearly dependent PMPL response of HMA at separate scattering band (650 nm) and absorption band (780 nm) as the function of laser intensity.

Figure S3 shows that PM-PL spectra of HMA (Diameter = 120 nm and Period = 360 nm) were recorded as the function of incident laser intensity. The perfect linear fits of PMPL peak as the function of laser power are reported in corresponding separate scattering (650 nm) and absorption band (780 nm) as shown in figure S4 (b), this clearly confirms the linear

nature of obtained PM-PL spectra in figure 2 of the manuscript.

Transient Response of 160nm diameter Hyperbolic Metaantenna

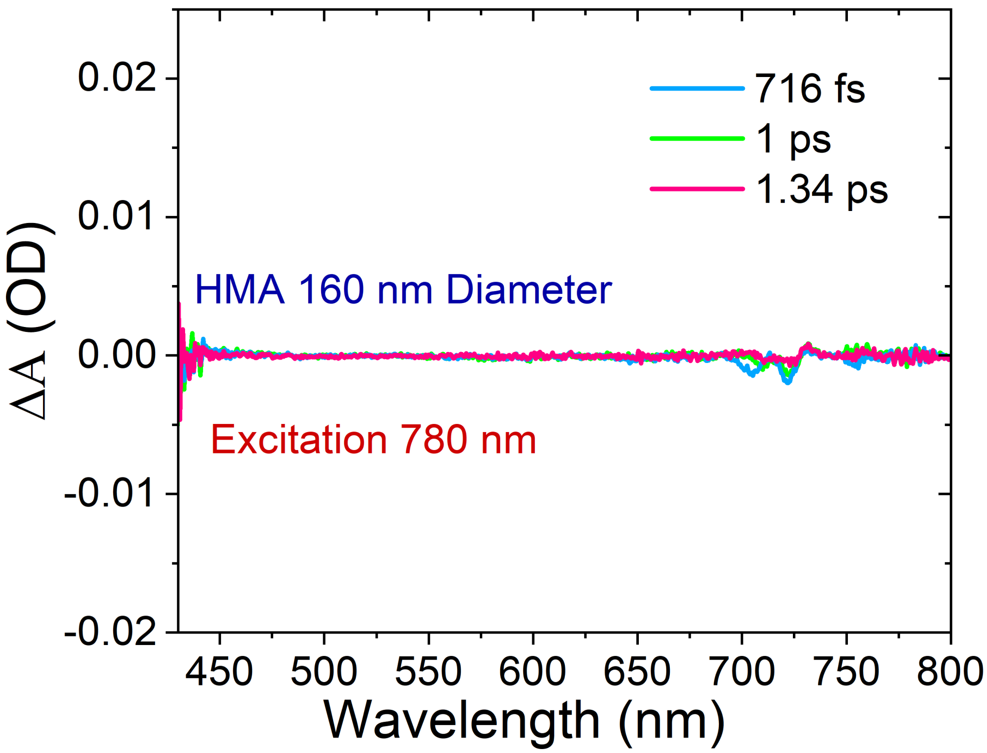

Figure S4: Transient response of 160 nm diameter HMA at specific time delays, when excited by 780 nm NIR pump pulses

HMA (160 nm diameter) exhibits the absorption resonance band around 950 nm. Therefore, Figure S4 shows no transient response on HMA (160 nm diameter) due to low absorption of HMA at 780 nm wavelength region, when excited by NIR pump pulses. This confirms that HEs can only be excited when pumped at the separate absorption resonance band of HMA.

References

(1) Johnson, P. B.; Christy, R.-W. Optical constants of the noble metals. *Physical review B* **1972**, *6*, 4370.

(2) Malitson, I. Interspecimen comparison of the refractive index of fused silica. *Josa* **1965**, *55*, 1205–1209.

(3) Shekhar, P.; Atkinson, J.; Jacob, Z. Hyperbolic metamaterials: fundamentals and applications. *Nano convergence* **2014**, *1*, 14.
